# Gastrin-releasing peptide receptor expression in non-cancerous bronchial epithelia is associated with lung cancer: a case-control study

**DOI:** 10.1186/1465-9921-13-9

**Published:** 2012-02-01

**Authors:** Ann Marie Egloff, Autumn Gaither Davis, Yongli Shuai, Stephanie Land, Joseph M Pilewski, James D Luketich, Rodney Landreneau, York E Miller, Jennifer R Grandis, Jill M Siegfried

**Affiliations:** 1 Department of Otolaryngology, University of Pittsburgh, Pittsburgh, Pennsylvania, USA; 2 Department of Medical Pharmacology and Chemical Biology, University of Pittsburgh, Pittsburgh, Pennsylvania, USA; 3 Department of Biostatistics, University of Pittsburgh Cancer Institute, Pittsburgh, Pennsylvania, USA; 4 Department of Medicine, University of Pittsburgh, Pittsburgh, Pennsylvania, USA; 5 Department of Surgery, University of Pittsburgh, Pittsburgh, Pennsylvania, USA; 6 Department of Medicine, Denver Veterans Affairs Medical Center, University of Colorado, Denver, Colorado, USA

**Keywords:** Gastrin-releasing peptide receptor, lung cancer risk, case-control study, surrogate tissue

## Abstract

**Background:**

Normal bronchial tissue expression of *GRPR*, which encodes the gastrin-releasing peptide receptor, has been previously reported by us to be associated with lung cancer risk in 78 subjects, especially in females. We sought to define the contribution of *GRPR *expression in bronchial epithelia to lung cancer risk in a larger case-control study where adjustments could be made for tobacco exposure and sex.

**Methods:**

We evaluated *GRPR *mRNA levels in histologically normal bronchial epithelial cells from 224 lung cancer patients and 107 surgical cancer-free controls. Associations with lung cancer were tested using logistic regression models.

**Results:**

Bronchial *GRPR *expression was significantly associated with lung cancer (OR = 4.76; 95% CI = 2.32-9.77) in a multivariable logistic regression (MLR) model adjusted for age, sex, smoking status and pulmonary function. MLR analysis stratified by smoking status indicated that ORs were higher in never and former smokers (OR = 7.74; 95% CI = 2.96-20.25) compared to active smokers (OR = 1.69; 95% CI = 0.46-6.33). *GRPR *expression did not differ by subject sex, and lung cancer risk associated with *GRPR *expression was not modified by sex.

**Conclusions:**

*GRPR *expression in non-cancerous bronchial epithelium was significantly associated with the presence of lung cancer in never and former smokers. The association in never and former smokers was found in males and females. Association with lung cancer did not differ by sex in any smoking group.

## Background

Lung cancer incidence rates have been declining for men since the 1980s. However, incidence rates for women over 65 have been increasing or have remained steady during this same time period [1]. Though tobacco use is a significant risk factor for lung cancer, 15-20% of lung cancer patients are lifetime never-smokers. Our group previously reported that bronchial epithelium expression of *GRPR*, which encodes the gastrin-releasing peptide receptor (GRPR), was associated with a diagnosis of lung cancer in female never smokers [2]. *GRPR *is an X-linked gene that has been reported to escape X-inactivation [3]. This finding raised the possibility that increased *GRPR *expression in women accounted for some of the increased incidence rates of lung cancer in never smokers who are female, compared to never smoking men, which was recently reported in a large prospective cohort study [4]. Since GRPR stimulation induces proliferative effects in bronchial cells [5], it is possible that activation of this pathway is a risk factor for lung cancer separate from that of tobacco exposure.

*GRPR *is overexpressed in lung cancers and in head and neck squamous cell carcinoma (HNSCC) [6,7]. We have previously reported elevated levels of *GRPR *mRNA in lung cancers and HNSCC [6,8]. In addition to cancer-specific overexpression of *GRPR*, we have demonstrated that mucosal tissues adjacent to HNSCC have *GRPR *mRNA levels reflective of the adjacent HNSCC tumor [6]. These findings suggest that elevated *GRPR *mRNA in normal bronchial epithelia may be associated with lung cancer risk and/or may indicate the presence of lung cancer.

We undertook a case-control study to determine whether elevated *GRPR *mRNA expression in normal, at-risk epithelium correlated with the presence of lung cancer. We evaluated the association between *GRPR *mRNA expression in purified cultured normal bronchial epithelial cells and the presence of lung cancer. Our primary finding was the observed increased expression of *GRPR *in normal bronchial epithelia in lung cancer cases compared to cancer-free controls. The impact of this was highest in subjects who never smoked or who had undergone smoking cessation before diagnosis. The association was found in both male and female never smokers, suggesting *GRPR *plays a similar role in development of lung cancer in men and women. The result of this study highlights *GRPR *overexpression in normal epithelial mucosa as a candidate risk factor for lung cancer, especially in those with limited tobacco exposure.

## Methods

### Lung cancer case-control study subjects and tissues

Lung cancer cases (n = 224) and surgical control subjects (n = 107) enrolled in prospective thoracic surgical tissue collection protocols from 1992-2004 donated mainstem bronchus biopsy specimens obtained at the time of resection, bronchoscopy, or lung transplant. Questionnaire and pulmonary function tests were administered prior to surgery, and forced expiratory volume in the first second (FEV1) and forced vital capacity (FVC) were used to assess airway obstruction [9]. Participants were patients with suspected lung cancer who underwent bronchoscopic or thoracic procedures. Cases had confirmed diagnoses of primary lung cancer while controls had non-cancerous diagnoses. Diagnoses occurring in 5% or more of surgical control subjects included the following: 15 had no diagnosis of disease (14%), 12 had emphysema (11%), 12 had a granuloma (11%), 11 had alpha-1 antitrypsin deficiency (10%), 7 had pulmonary hypertension (9%), 7 had a benign growth (7%), 6 had a lung obstruction (6%), 5 had cystic fibrosis (5%), 5 had a hamartoma (5%) and 5 had pulmonary fibrosis (5%). Two of the 15 control subjects with no diagnosis of disease were lung donors. Tissues from 219 of the 224 cases and 89 of the 107 controls were prospectively collected under protocols approved by the University of Pittsburgh institutional review board (IRB). In a cooperative effort, tissues from 5 of the 224 cases and 18 of the 107 controls were prospectively collected under a surveillance bronchoscopy protocol approved by the University of Colorado IRB. The case-control study populations are described in Table 1. Primary bronchial epithelial cell culture procedures were used to obtain proliferating bronchial epithelial cells as described previously [5]. Bronchial epithelial cell cultures were harvested at passage 1 for *GRPR *mRNA expression studies.

**Table 1:** Characteristics of Lung Cancer Cases and Controls

Characteristic	Lung Cancer Cases (N = 224)		Cancer-free Controls (N = 107)		P
Age, y					
Median (Range)	68 (40-85)		52 (31-83)		< 0.001^†^
Sex, N %					
Male	105	46.9%	60	56.1%	0.12^‡^
Female	119	53.1%	47	43.9%	
Ethnicity, N %					
Caucasian	212	95.5%	101	94.4%	0.70^§^
African-American	9	4.1%	5	4.7%	
Other	1	0.5%	1	0.9%	
Smoking Status, N %					
Never smoker	19	8.5%	33	30.8%	< 0.001^‡^
Ex-smoker	111	49.6%	57	53.3%	
Active smoker	87	38.8%	14	13.1%	
Unknown	7	3.1%	3	2.8%	
Pack-Years					
Median (Range)	50 (0-150)		25 (0-156)		< 0.001^†^
Unknown	0-150		0-156		
Lung Function, N %					
Normal	89	39.7%	36	33.6%	< 0.001^§^
Mild Obstruction	14	6.3%	5	4.7%	
Moderate Obstruction	52	23.2%	16	15.0%	
Severe Obstruction	37	16.5%	46	43.0%	
Unknown	32	14.3%	4	3.7	
Tumor Type, N %					
Adenocarcinoma	112	50.0%	-		
Squamous cell carcinoma	60	26.8%	-		
Other NSCLC	27	12.1%	-		
Small cell lung cancer	10	4.5%	-		
Other/unknown	15	6.7%	-		
Stage, N %					
TIS-I	99	44.2%	-		
II	38	17.0%	-		
III	49	21.9%	-		
IV	16	7.1%	-		
Not Staged	22	9.8%	-		
Follow-up, N %					
Alive	78	34.8%	-		
Dead	142	63.4%	-		
Median survival, months^#^	35.8		-		
(95% CI)	(31.7-40.5)		-		

^#^Median overall survival for cancer cases alive at last follow-up

^†^Rank sum test

^‡^Chi-square test

^§^Fisher's exact test

### Detection of *GRPR *expression in bronchial epithelial cells

RNA isolation from bronchial cells and detection of *GRPR *gene expression was performed as previously described [5]. PCR amplification was performed following oligo-dT-primed reverse transcription (RT) of total RNA using primers GRPR-1 (5'-CTCCCCGTGAACGATGACTGG-3') and GRPR-2 (5'-ATCTTCATCAGGGCATGGGAG-3'). Presence of GRPR product was evaluated by hybridization with a ^32^P-labeled internal probe (5'-CACCTCCATGCTCCACTTTGTC-3'). The *GAPDH *gene expression was also evaluated in order to assess RNA integrity and success of the reverse transcription step. GAPDH was successfully amplified from RNA isolated from all cases and controls.

### Statistical Analyses

In order to test the association of sex and other variables with *GRPR *bronchial expression, candidate confounding variables including age, sex, ethnicity, smoking status, pack-years of tobacco-use (py), and pulmonary function were evaluated for association with *GRPR *expression separately for cases and controls for each case-control study using the chi-square test, Fisher's exact test, or Wilcoxon rank sum test as appropriate. All P values reported were 2-sided with significance defined by P < 0.05.

Evaluating *GRPR *expression in non-cancerous bronchial epithelia among cases versus controls was the primary endpoint of the study. Univariate and multivariable logistic regression models were implemented to assess the significance of the association of *GRPR *bronchial expression with cancer before and after controlling for other important variables. For these models, age and sex were defined *a priori *to be included. Sex, ethnicity and smoking status were treated as categorical variables, age and pack-years as continuous variables, and pulmonary function as an ordinal variable. The likelihood ratio test was used to test the goodness of fit of logistic regression models. To assess whether the association between bronchial *GRPR *expression and lung cancer was modified by sex, a sex by *GRPR *expression interaction term was evaluated for significance in the multivariable logistic regression model. Because disease etiology likely differs for never smokers compared to smokers, a stratified analysis by smoking status was also performed.

Association of *GRPR *expression with overall survival, defined as time from surgery to death, was analyzed using Cox proportional hazards models. Date of surgery, death, and last follow-up were provided by the University of Pittsburgh Cancer Institute (UPCI) Lung Cancer Registrar. Hazard ratios associated with *GRPR *expression were estimated using multivariable Cox proportional hazards models. The assumption of proportional hazards was assessed for all Cox models by evaluation of scaled Schoenfeld residuals.

## Results

### GRPR expression in bronchial epithelia was more frequent in lung cancer patients than cancer-free control subjects

Presence or absence of *GRPR *mRNA in non-cancerous bronchial epithelial cells derived from mainstem bronchus airway biopsies of lung cancer cases (n = 224) and cancer-free controls (n = 107) (Table 1) was assessed by RT-PCR followed by hybridization with a radio-labeled probe in order to maximize sensitivity. Because the airway biopsy analysis of this cohort began before quantitative PCR (q-PCR) was available, the RT-PCR semi-quantitative technique was used throughout the lung cancer case-control study to maintain consistency and power. Of the 224 lung cancer cases evaluated, 158 (71%) had *GRPR *expression in bronchial cells. In contrast, only 41 (38%) of the 107 cancer-free surgical controls had detectable *GRPR *bronchial expression.

*GRPR *expression has been reported to be elevated with tobacco use. We tested for association between *GRPR *expression in non-cancerous surrogate tissues and smoking status and pack-years of tobacco use stratified by cancer status. Among never and former smokers, lung cancer patients more frequently had detectable *GRPR *expression while the frequency of detected *GRPR *mRNA was similar for actively smoking lung cancer cases and controls (Table 2). We observed a statistically significant association between *GRPR *expression in bronchial mucosa and smoking status among lung cancer patients (P = 0.03, Table 2) with overrepresentation of *GRPR *bronchial expression among never smoking lung cancer patients. *GRPR *bronchial expression was also associated with smoking status among cancer-free surgical controls with an overrepresentation of *GRPR *bronchial expression among active smokers (P = 0.02) (Table 2). Only a minority of the never smoker and former smoker surgical cancer-free control subjects had *GRPR *bronchial expression while, the majority of actively smoking surgical controls had detectable *GRPR *bronchial expression. Though there was a significant association between *GRPR *expression and smoking status for cases and controls, in the analyses stratified by case status we found no statistically significant association between pack-years of smoking and *GRPR *expression in cases or controls (Table 2).

**Table 2:** Evaluation of association between GRPR broncial expression and demographic and risk factors stratified by lung cancer case status.

	Lung Cancer Cases						Controls					
Characteristic	Total	GRPR Positive		GRPR Negative		P	Total	GRPR Positive		GRPR Negative		P
N	224	158	70.5%	66	29.5%		107	41	38.3%	66	61.7%	
Age												
Median (Range)	224	67 (42-84)		70 (41-85)		0.01*^†^		52 (31-80)		52 (31-83)		0.44^†^
Sex												
Male	105	75	71.4%	30	28.6%	0.78^‡^	60	20	33.3%	40	66.7%	0.23^‡^
Female	119	83	69.7%	36	30.3%		47	21	44.7%	26	55.3%	
Ethnicity												
Caucasian	213	150	70.4%	63	29.6%	1.00^§^	101	38	37.6%	63	62.4%	0.67^§^
Non-Caucasian	10	7	70.0%	3	30.0%		6	3	50.0%	3	50.0%	
Smoking Status												
Never Smoker	19	17	89.5%	2	10.5%	0.03*^§^	33	11	33.3%	22	66.7%	0.02*^§^
Former Smoker	111	70	63.1%	41	36.9%		57	18	31.6%	39	68.4%	
Active Smoker	87	65	74.7%	22	25.3%		14	10	71.4%	4	28.6%	
Pulmonary Function												
Normal	89	62	69.7%	27	30.3%	0.52^§^	36	18	50.0%	18	50.0%	0.22^§^
Mild Obstruction	14	9	64.3%	5	35.7%		5	2	40.0%	3	60.0%	
Moderate Obstruction	52	37	71.2%	15	28.8%		16	5	31.3%	11	68.8%	
Severe Obstruction	37	30	81.1%	7	18.9%		46	13	28.3%	33	71.7%	
Pack-Years												
Median (Range)	222	50 (0-150)		50 (0-120)		0.94^†^		40 (0-110)		25 (0-156)		0.11^†^
Disease Stage												
CIS	1	1	100.0%	0	0.0%	0.49^§^	-	-		-		-
1	98	69	70.4%	29	29.6%		-	-		-		
2	38	24	63.2%	14	36.8%		-	-		-		
3	49	39	79.6%	10	20.4%		-	-		-		
4	16	11	68.8%	5	31.3%		-	-		-		
Histology												
Adenocarcinoma	112	74	66.1%	38	33.9%	0.50^§^	-	-		-		-
Squamous cell carcinoma	60	47	78.3%	13	21.7%		-	-		-		
Other NSCLC	27	19	70.4%	8	29.6%		-	-		-		
Small cell lung cancer	10	8	80.0%	2	20.0%		-	-		-		
Other/unknown	15	10	66.7%	5	33.3%		-	-		-		

*Significant at P < 0.05

^† ^Wilcoxon rank sum test

^‡ ^Chi-square test

^§ ^Fisher's exact test

### Bronchial GRPR expression was not associated with sex, ethnicity or pulmonary function in lung cancer cases or controls

In order to assess the association of subject characteristics with *GRPR *expression independent of cancer, a stratified analysis by case status was performed separately for the lung cancer case and control populations. We hypothesized that *GRPR *expression would differ by sex because *GRPR *resides on the portion of the X chromosome reported to escape X-inactivation [3], and we had previously reported that *GRPR *expression in bronchial tissues was more frequent in women never smokers than men never smokers in a study involving 78 subjects [2].

In this larger study involving 331 subjects, in addition to evaluating differences in *GRPR *expression by sex, we also tested the association between *GRPR *expression and age, ethnicity and pulmonary function. *GRPR *bronchial expression levels did not differ by sex, ethnicity or pulmonary function for either lung cancer cases or controls (Table 2). However, lung cancer cases positive for *GRPR *expression were statistically younger than GRPR negative cases (Table 2).

Evaluating the *GRPR *expression distribution among surgical cancer-free controls by benign diagnosis, we observed a trend towards a significant association (p = 0.065, Fisher's exact test) with deviations from the average 38% GRPR positive frequency being most apparent for diagnoses of alpha-1 trypsin deficiency (1 of 11 subjects were GRPR positive) and granuloma (9 of 12 subjects were GRPR positive). Only two of the 9 GRPR positive granuloma subjects were active smokers. This trend towards increased bronchial *GRPR *expression in subjects with granuloma that did not reach statistical significance suggests the possibility that inflammation-induced tissue damage and/or wound repair in response to damage may be associated with bronchial *GRPR *expression. We did not find evidence of an association between bronchial GRPR expression and hyperproliferative disorders among the cancer-free control subjects.

### GRPR expression levels did not reflect disease stage or tumor type

In order to determine whether *GRPR *expression differed by tumor clinical and/or pathological characteristics, we tested for association between tumor characteristics and bronchial *GRPR *expression in lung cancer cases. The distribution of lung cancer cases by clinical stage and tumor histology is provided in Table 1. Bronchial *GRPR *expression in non-cancerous mucosa was independent of disease stage (P = 0.49, Table 2) and tumor histology (P = 0.50, Table 2).

### GRPR expression in non-cancerous bronchial mucosal tissues was significantly associated with lung cancer independent of age, sex and smoking status

**Figure 1:**
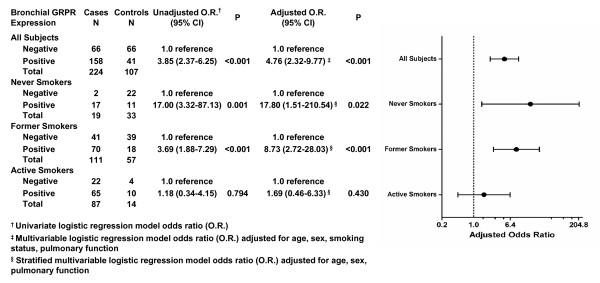
**GRPR expression in bronchial epithelia is significantly associated with lung cancer among never and former smokers**. Univariate and multivariable logistic regression analysis estimates of odds ratios are provided for all subjects, and separate analyses are provided by smoking status.

Detection of *GRPR *mRNA in bronchial tissues was significantly associated with presence of lung cancer (O.R = 3.85; 95% CI = 2.37-6.25) (All Subjects, Figure 1). Even after controlling for possible confounding effects of age, sex, smoking status and pulmonary function expression of *GRPR *in normal bronchial epithelium remained significantly associated with lung cancer (O.R. = 4.76; 95% CI = 2.32-9.77) (All Subjects, Figure 1). *GRPR *was previously reported by our group in a study of 78 subjects to be more frequently expressed in women with lung cancer than in men with lung cancer [2], suggesting the possibility that this differential expression may account at least in part for the heightened smoking-related lung cancer risk for women observed in some studies [10,11]. However, in this larger study, the pair-wise interaction between sex and *GPRR *expression was found not to be significant (P = 0.31) in a multivariable logistic regression model also containing age, sex, pulmonary function and bronchial *GRPR *expression. Therefore, this study did not support our previous hypothesis that *GRPR *expression among women contributed to their increased lung cancer risk. Rather, it suggested that never smoking status was a confounder in our previous analysis, since the majority of never smokers diagnosed with lung cancer are female.

### GRPR expression in bronchial epithelium was significantly associated with lung cancer among never smokers and former smokers but not active smokers

Molecular alterations differ markedly for tobacco-related versus non-tobacco-related lung cancers [12,13]. These finding indicate that disease etiology for lung cancer varies by tobacco-use history. A stratified analysis by smoking status indicated that presence of *GRPR *expression in bronchial cells was significantly associated with lung cancer for never smokers (O.R. = 17.0; 95% C.I = 3.32-87.13) and former smokers (O.R. = 3.69; 95% C.I = 1.88-7.29) but not active smokers (O.R. = 1.18; 95% CI = 0.34-4.15) (Figure 1), and these odds ratios differed significantly (P < 0.001, Mantel-Haenszel test of homogeneity (M-H test)). The association between *GRPR *bronchial expression and lung cancer among never smokers and former smokers was significant even after controlling for the effects of age, sex, and pulmonary function (Figure 1). The combined, adjusted odds ratio for never and former smokers, which were found to not differ significantly (P = 0.09; M-H test), was 7.74 (95% CI = 2.96-20.25). A trend towards higher odds ratios with fewer pack-years of tobacco use was observed when the analysis was stratified by tertiles of control subject pack-year use (data not shown); however, the odds ratios did not differ significantly by tertile pack-year category.

Because we hypothesized that *GRPR *expression in bronchial epithelia would contribute to increased risk of lung cancer for women never smokers compared to men never smokers, we tested the interaction between sex and *GRPR *expression status for association with lung cancer in the never smoking stratum. We found the *GRPR *expression by sex interaction to not be significantly associated with lung cancer among never smokers in a multiple logistic regression model also containing age, sex and pulmonary function (P = 0.94). Because the number of never smokers in our study was not large (n = 52) and the association between *GRPR *expression and lung cancer did not differ significantly between never and former smokers, we also tested the *GRPR *expression by sex interaction for significance for never and former smokers. We found that the *GRPR *expression by sex interaction was also not significant in this same model when evaluating never and former smokers (P = 0.47).

### GRPR expression in surrogate tissues was not a prognostic indicator for survival in lung cancer cases

Because *GRPR *expression in bronchial epithelia was associated with lung cancer, we hypothesized that for cases with detectable bronchial *GRPR *expression, survival would be reduced compared to cases without detectable *GRPR *expression. However, the presence of *GRPR *mRNA in bronchial cells was not an indication of overall lung cancer survival. The hazards ratio for overall lung cancer survival in Cox models adjusted for age, sex, and disease stage was 0.87 (95% CI = 0.57-1.34) for patients with *GRPR *bronchial expression compared to patients without detectable *GRPR *bronchial expression.

## Discussion

In lung cancer GRPR and its ligand are involved in autocrine growth stimulation [6,14]. Previous results showed that lung tumor tissues have elevated *GRPR *expression, and we have previously described elevated *GRPR *mRNA levels in histologically normal mucosal tissues adjacent to HNSCC compared to oral mucosal tissues from cancer-free control subjects [6]. *GRPR *mRNA expression has also been detected in prostate tumors and tissues adjacent to prostate cancers [15]. Our new findings reported here indicate that in a prospectively collected lung cancer case-control population, *GRPR *expression in at-risk upper aerodigestive mucosa was significantly associated with lung cancer. Importantly, even after controlling for the effects of possible confounding by age, sex, and tobacco use, *GRPR *expression in non-cancerous mucosal tissues was significantly associated with lung cancer among never and former smokers and appeared to confer similar risk to both sexes.

Our finding here of no difference in bronchial epithelial cell *GRPR *expression between men and women did not replicate our previous result [2]. In our previous bronchial cell study, which had a smaller sample size of 78 patients, the presence of cancer was not separately evaluated, and only one never smoker with lung cancer was male [2]. In retrospect, it is likely that the associations between *GRPR *expression, female sex, and smoking observed in our previous study were actually surrogates for the underlying association between bronchial epithelial *GRPR *expression and lung cancer, which appears to be most significant in never smokers. Therefore, this study presents important revisions to our previous understanding of the role of *GRPR *in lung cancers arising in females and males; this study supports a similar role for bronchial *GRPR *mRNA expression and lung cancer risk for both females and males.

Although in the current study we also found no association between *GRPR *expression and pack-years of smoking, we did observe associations between smoking status (active versus former smoker versus never smoker) and *GPRR *expression. We observed these associations for both cases and controls separately, but the relationships were different. The proportion of *GRPR *positive actively smoking cancer-free control subjects was similar to the proportion of actively smoking lung cancer cases. These findings were consistent with our previously reported findings that *GRPR *expression in bronchial epithelium was activated with tobacco use [2] and with findings that bombesin-like peptide receptors play a role in wound healing following airway injury [16]. Similar to our previous findings of persistent bronchial *GRPR *expression after tobacco cessation [5], we detected bronchial *GRPR *expression in the majority of former smoking lung cancer cases. In contrast, in the current study bronchial *GRPR *expression was detected in only a minority of cancer-free controls who were former smokers. The inclusion of more cancer-free control subjects in this study compared to our 1997 study has allowed us to evaluate lung cancer patients and cancer-free controls separately and has revealed new insights regarding the relationship between bronchial *GRPR *expression and tobacco use in cancer-free controls.

Although the number of active smoking surgical controls in our current study was small, the data suggest that bronchial *GRPR *expression may be induced by tobacco use in subjects without lung cancer, but that the increase in *GRPR *expression in bronchial mucosa likely subsides following the cessation of smoking in most subjects without lung cancer. Among former smoking lung cancer cases, bronchial *GRPR *expression may be aberrantly maintained following cessation of smoking, or similar to never smoking lung cancer cases, bronchial *GRPR *expression may reflect risk that is independent of tobacco use. Our 1997 report indicated that of the 4 cancer-free subjects with defined bronchial *GRPR *expression and smoking status, 1 active smoker was GRPR positive while 3 subjects who were former or never smokers were negative for *GRPR *expression. Therefore, although the numbers are small, among cancer-free control subjects, the relationship between smoking status and bronchial *GRPR *expression in the 1997 study is consistent with our current study results.

Of special interest was the finding of frequent bronchial *GRPR *expression among never smoking lung cancer cases. While GRPR expression was detected in only a minority of never smoking cancer-free controls, GRPR expression was detected in almost 90% of never smoking lung cancer cases. Though the specific cause of GRPR expression in never smoking lung cancer cases is unknown, we posit that bronchial GRPR expression may reflect an inherent or conferred risk factor that can be best observed in the absence of the more potent risk factor of tobacco use. Though bronchial G*RPR *expression was more common among cancer-free controls with a diagnosis of granuloma, suggesting a possible inflammatory component to bronchial *GRPR *expression among cancer-free controls, bronchial *GRPR *expression was not increased in lung cancer cases or controls with more severe pulmonary obstruction, which also has an inflammatory component. Therefore, the role of inflammation in elevated bronchial *GRPR *expression remains undefined.

*GRPR *expression in normal bronchial tissues was not correlated with clinical disease stage. Therefore, our data suggest that *GRPR *expression in surrogate tissues did not reflect tumor burden and, perhaps, was not a direct consequence of the prevalent cancer. Our finding that detectable *GRPR *expression in normal upper aerodigestive tissues was not an indication of poor overall survival for lung cancer cases indicates that elevated *GRPR *bronchial cell expression was not associated with disease progression and is, instead, likely to be a marker of risk exposure or a marker of host susceptibility.

Lung cancer cases positive for *GRPR *bronchial expression were significantly younger than cases negative for *GRPR *expression, which supports the role of *GRPR *bronchial expression as conferring lung cancer risk. Though a prospective cohort study will be required to fully understand the relationship between *GRPR *expression levels in surrogate tissues and the development of lung cancer, *GRPR *expression in normal bronchial tissues has potential value as a marker for elevated risk, especially in those with little or no tobacco exposure. Though *GRPR *is overexpressed in many solid tumors, only one other group has evaluated *GRPR*, *GRP *and/or their gene product levels in surrogate tissues of cancer patients to date. Uchida et al. reported that serum levels of proGRP, as measured by enzyme-linked immunosorbent assay (ELISA), correlated with tumor *GRP *gene expression levels in small cell lung cancer (SCLC) patients [17]. *GRPR *expression levels in tumors were not evaluated in our study, as material for analysis was not available.

The increased risk due to elevated *GRPR *expression may be most apparent in never and former smokers because the contribution of *GRPR *expression to risk is obscured in active smokers by factors such as genetic abnormalities and inflammatory processes that confer substantial risk from tobacco use. Elevated *GRPR *expression in the lung may independently contribute to increased cancer risk by promoting proliferation. *GRPR *is expressed at early embryonic stages in the nervous, urogenital, respiratory, and gastrointestinal systems and expression in these tissues is generally down-regulated before birth [18-20]. The GRPR ligand, GRP, a bombesin-like peptide (BLP) growth factor, is expressed by pulmonary neuroendocrine cells and has been shown to stimulate lung development in utero and to increase growth and maturation of human fetal lung organ cultures [20,21]. In non-cancerous tissues, BLPs stimulate growth of bronchial, gastrointestinal and pancreatic epithelial cells and lead to ligand-dependent hyperplasia [5,19,22-24]. GRPR and GRP are involved in an autocrine stimulation loop in lung cancer and HNSCC [6,14], and *GRPR *expression has been shown to be positively regulated by GRP [20]. Increased *GRPR *expression in the lung may, therefore, reflect a state that is more nascent and proliferative in nature than epithelium with low or undetected *GRPR *expression.

We acknowledge our case-control study population limitations. This case-control study required hospital surgical controls, and this limited recruitment with the result that our lung cancer cases are older than our controls and include fewer men. In addition, the exhaustion of samples made quantitative measurements of *GRPR *mRNA expression impossible. We have confined our analysis to GRPR mRNA because of antibody reagent limitations at the time the samples were evaluated. This leaves the question of whether GRPR protein levels also differ unanswered. Despite these limitations, a high degree of association between detectable *GRPR *expression in normal bronchial tissue and lung cancer was demonstrated in the case-control population even after adjusting for sex and age.

Though we did not assess the epidermal growth factor receptor (*EGFR*) mRNA or protein expression in bronchial epithelial cultures, we speculate that increased *GRPR *expression contributes to lung cancer through EGFR-dependent and/or -independent mechanisms. The EGFR pathway has been reported to be activated in lung tumors from never smokers with EGFR mutations, and it is possible that lung tumors developing in never smokers have multiple mechanisms for EGFR activation. The GRPR pathway is known to interact with the EGFR pathway in lung cancer cells by increasing the release of EGFR ligands such as amphiregulin [8], which could act to further promote cancer in never smokers who develop EGFR mutations. Alternatively, activation of the GRPR pathway may increase EGFR bronchial cell signaling in the absence of EGFR mutation, providing another route to lung cancer development in never smokers.

## Conclusions

The GRPR pathway may activate proliferative pathways that increase the likelihood of lung cancer development in male and female former and never smokers. We conclude from our data that *GRPR *expression likely does not contribute to sex differences in rates of lung cancer incidence in never or former smokers.

## List of Abbreviations

CI: confidence interval; FEV1: forced expiratory volume in the first second; FVC: forced vital capacity; GRPR: gastrin-releasing peptide receptor; HNSCC: head and neck squamous cell carcinoma; M-H: test Mantel-Haenszel test of homogeneity; OR: odds ratio

## Competing interests

The authors declare that they have no competing interests.

## Authors' contributions

AME contributed to the conception of analyses, performed analyses, interpreted statistical analyses and writing of the manuscript. AGD coordinated sample storage, retrieval and database. YS and SL performed statistical analyses. JMP, JDL, RD, YEM enrolled patients into the clinical trial, collected and provided specimens for analysis. JRG contributed to the conception and design of this project and contributing to the writing of this manuscript. JMS contributed to the conception and design of this project and contributed to the writing of this manuscript. All authors have read and approved the final manuscript
